# Association Between Institutional Social Media Involvement and Gastroenterology Divisional Rankings: Cohort Study

**DOI:** 10.2196/13345

**Published:** 2019-09-06

**Authors:** Austin Lee Chiang, Loren Galler Rabinowitz, Akhil Kumar, Walter Wai-Yip Chan

**Affiliations:** 1 Brigham and Women's Hospital Boston, MA United States; 2 Harvard Medical School Boston, MA United States; 3 Columbia University Medical Center New York, NY United States; 4 Washington University in St Louis St. Louis, MO United States

**Keywords:** social media, Twitter, hospital ranking

## Abstract

**Background:**

Patients often look to social media as an important tool to gather information about institutions and professionals. Since 1990, United States News and World Report (USNWR) has published annual rankings of hospitals and subspecialty divisions. It remains unknown if social media presence is associated with the USNWR gastroenterology and gastrointestinal (GI) surgery divisional rankings, or how changes in online presence over time affects division ranking.

**Objective:**

The objective of this study was to determine if social media presence is associated with USNWR gastroenterology and GI surgery divisional rankings and to ascertain how changes in online presence over time affect division rankings.

**Methods:**

Social media presence among the top 30 institutions listed in the 2014 USNWR gastroenterology and GI surgery divisional rankings were assessed using Pearson’s correlation coefficients and multivariate analysis, controlling for covariates. Linear and logistic regression using data from 2014 and 2016 USNWR rankings were then used to assess the association between institutional ranking or reputation score with any potential changes in numbers of followers over time. Sensitivity analysis was performed by assessing the area under the receiver operating characteristic curve to determine the follower threshold associated with improved or maintained ranking, which was done by dichotomizing changes in followers at values between the 7000 and 12,000 follower mark.

**Results:**

Twitter follower count was an independent predictor of divisional ranking (β=.00004; *P*<.001) and reputation score (β=–.00002; *P*=.03) in 2014. Academic affiliation also independently predicted USNWR division ranking (β=5.3; *P*=.04) and reputation score (β=–7.3; *P*=.03). Between 2014 and 2016, Twitter followers remained significantly associated with improved or maintained rankings (OR 14.63; 95% CI 1.08-197.81; *P*=.04). On sensitivity analysis, an 8000 person increase in Twitter followers significantly predicted improved or maintained rankings compared to other cutoffs.

**Conclusions:**

Institutional social media presence is independently associated with USNWR divisional ranking and reputation score. Improvement in social media following was also independently associated with improved or maintained divisional ranking and reputation score, with a threshold of 8000 additional followers as the best predictor of improved or stable ranking.

## Introduction

Social media refers to a variety of web-based platforms where individuals create and share information, experiences, and ideas. In recent years, social media has transformed into an important form of communication that has led to significant changes across the globe, including political uprisings. The Office of Health Information Technology within the United States Department of Health and Human Services has recognized the significance of social media in healthcare and the opportunities that lie therein for both patients and providers [1]. Medical professionals and organizations utilize social media to promote health through education and advocacy, announce news and events, recruit employees, and communicate with other providers [2]. Similarly, patients use social media to gain medical knowledge, share experiences, and provide feedback [3]. A reported 24-42% of consumers have used social media to access health-related consumer reviews, including reviews of hospitals [3,4].

Twitter is one social media platform that allows individuals or organizations to post messages limited to 280 characters or less. According to Twitter’s official website, there are more than 300 million monthly active users around the world and over 500 million tweets per day [5]. Users may also follow the accounts of peers, public figures, and organizations, whose posts will automatically be displayed in a live, chronological feed. In recent years, more gastroenterologists have joined Twitter and engaged in academic discussions on a daily basis. Social media presence is modifiable and increased professional engagement could potentially impact institutional recognition and reputation. In a case study of a corporate brand, Twitter has been shown to amplify positive brand sentiments, and not only shape brand awareness and recognition but also reflect curiosity surrounding a brand [6]. Increased activity on Twitter has been shown to be correlated with Twitter following among US hospitals [7].

United States News and World Report (USNWR) boasts a reach of over 300 million readers [8]. Since 1990, USNWR annually publishes a Best Hospitals ranking of institutions and subspecialties, with 27.5% of an institution’s overall score determined by reputation score [6]. These rankings are often used as a marketing tool by hospital leaders to attract patients through traditional methods of advertising, as well as on social media [9-11]. USNWR hospital rankings have been shown to have a significant impact on consumer decisions in the form of a greater than 5% change in nonemergency Medicare patients, and an estimated $76 million in revenue transferred from lower ranked hospitals to higher ranked hospitals [9]. Among 42 urology departmental Twitter accounts, there was a significant correlation between Twitter followers with USNWR reputation score [12].

The aim of this study was to determine the degree to which institutional social media involvement, based on number of followers on Twitter, may be associated with USNWR gastroenterology and gastrointestinal (GI) surgery divisional rankings (all rankings henceforth are divisional rankings). A secondary aim was to determine whether social media presence is a predictor of improvement in USNWR rankings, and to attempt to define the growth necessary to achieve improved division rankings.

## Methods

This was a cohort study of the top 30 institutions listed in the 2014 and 2016 USNWR gastroenterology and GI surgery subspecialty rankings. Data from the 2014 rankings were used to address the first aim, and to address the secondary aims a follow-up study compared data based on the 2014 and the 2016 USNWR rankings. Both the numeric ranking and the institutional reputation score were publicly available on the USNWR webpage. The number of Twitter followers corresponding to the 2014 and 2016 USNWR rankings were accessed in June 2015 and November 2016 at time of analysis, respectively, as displayed on each institution’s Twitter account. The official institutional Twitter accounts were identified using standard internet search tools, and hospital-specific Twitter accounts were included. Twitter accounts that appeared to combine hospital with medical school affairs were included. Medical school–specific, or university-specific accounts distinct from a hospital-based Twitter account, were excluded.

Spearman’s rank correlation coefficient was calculated to evaluate the association between institutional ranking and social media following. Pearson’s correlation coefficients were calculated to determine the association between social media presence and divisional reputation score. Multivariate analysis using linear regression was performed, controlling for number of hospital beds, academic affiliation, city population, and the number of full-time gastroenterologists on staff. SAS software (Cary, North Carolina) was used for statistical analyses. Among the covariates, the number of hospital beds was available online as it was published in the USNWR rankings. Other covariates such as academic affiliation (defined as an associated teaching hospital with gastroenterology fellowship training program) and number of full-time gastroenterologists on staff were obtained from the institutions’ official websites. Local population size of the city in which the institution is located was obtained from the latest United States Census Bureau figures.

For our secondary aim, two-tailed, two-sample *t* tests were performed to assess the association between 2014 and 2016 institutional rankings, or reputation scores, with changes in numbers of followers between 2014 and 2016. Multivariate analysis was performed using logistic and linear regression to adjust for the aforementioned covariates. Sensitivity analysis was performed by assessing the area under the receiver operating characteristic curve (AUROC) to determine the follower threshold associated with improved or maintained ranking, which was done by dichotomizing changes in followers at the 7000, 8000, 9000, 10,000, and 12,000 follower mark.

## Results

**Figure 1:**
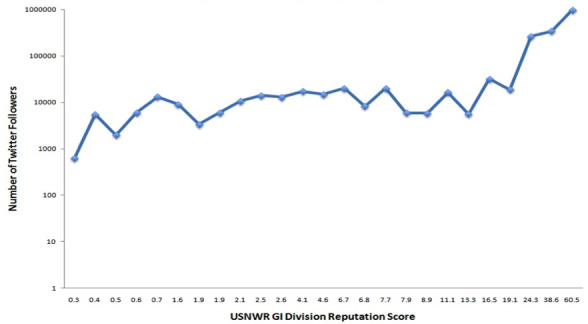
Twitter followers versus 2014 United States News and World Report gastrointestinal reputation score. GI: gastrointestinal. USNWR: United States News and World Report.

Of the top 29 institutions in the 2014 USNWR gastroenterology and GI surgery rankings, there were 28 distinct institutional Twitter accounts and one institution without a Twitter account (John Muir Medical Center). Among these Twitter accounts, the number of followers ranged from 0 to 1.7 million as of June 2015. Spearman’s rank correlation coefficient demonstrated a negative correlation between the number of Twitter followers and the numeric divisional ranking (ρ=–0.582; *P*<.001). Similarly, there was a positive correlation between Twitter followers and the divisional reputation score (ρ=0.91; *P*<.001) (Table 1). Twitter follower count was an independent predictor of divisional ranking on multivariate analysis controlling for the number of hospital beds, academic affiliation, local population, and number of full-time gastroenterologists on staff (β=0.00004, *P*<.001) (Table 1). Quantity of Twitter followers was also an independent predictor of USNWR reputation score after controlling for the same covariates (β=–0.00002, *P*=0.03) (Table 2 and Figure 1). Academic affiliation was an independent predictor of division ranking (β=5.3; *P*=0.04) and reputation score (β=–7.3; *P*=0.03) on USNWR (Table 2 and Table 3).

**Figure 2:**
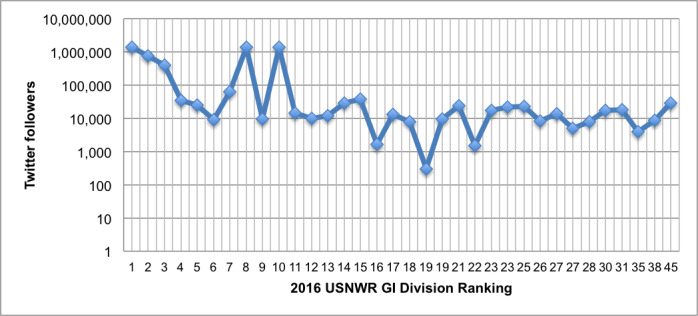
Twitter followers by 2016 United States News and World Report ranking. GI: gastrointestinal. USNWR: United States News and World Report.

**Figure 3:**
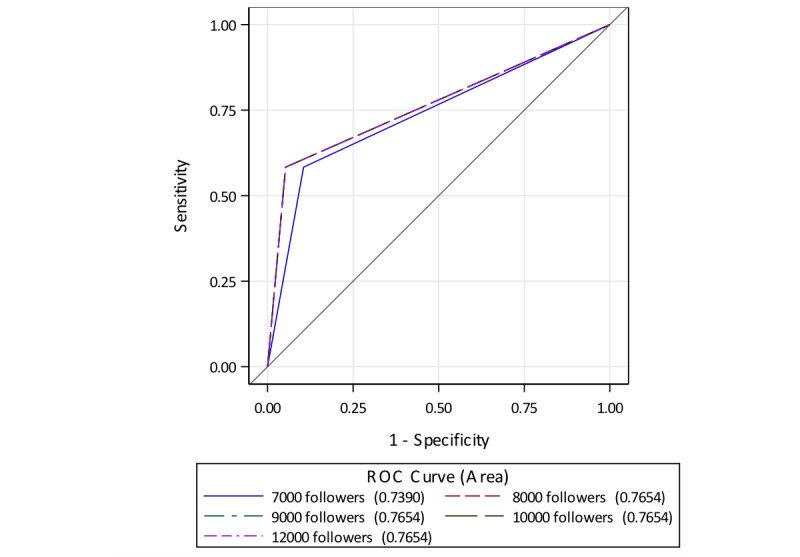
Receiver operating characteristic curve for different follower cutoffs. ROC: receiver operating characteristic.

For the second aim, 39 hospitals were included in the analysis (Figure 2). Of the 2014 ranked institutions, five hospitals were no longer ranked in 2016 and four had rankings that had slipped under 30. Ten hospitals were newly ranked in the top 30 gastroenterology and GI surgery programs, and there were two institutions that were tied at rank #23 and #27. Of note, by 2016 the Mayo Clinic appeared three times in the top 30 ranking despite there being separate entries for the Rochester (MN), Phoenix (AZ), and Jacksonville (FL) campuses. Overall, 12 hospitals showed improved or maintained institutional ranking. Institutions with improved or maintained rankings had a significantly higher increase in followers over 1 year, with a difference of 89,287 versus 2271 (*P*=.03). On logistic regression, change in Twitter followers remained significantly associated with improved or maintained rankings (OR 14.63; 95% CI 1.08-197.81; *P*=.04). On sensitivity analysis, an increase of 8000 Twitter followers was associated with significantly higher area under the curve (AUC) (0.765) for prediction of improved or maintained ranking compared to other cutoffs (Figure 3). On multivariate analyses of 2016 rankings controlling for potential confounders using linear regression, the current number of Twitter followers for an institution was significantly associated with higher rank (β=–0.000017; *P*=.03) and reputation score (β=0.000036; *P*<.001). Academic status was also a significant predictor (β=3.446; *P*=.03) for reputation score.

**Table 1:** Rankings and characteristics of ranked gastroenterology and GI^a^ institutions.

Hospital with Gastroenterology and GI Surgery Program	Rank 2014	Rank 2016	Followers 2014	Followers 2016	Reputation score 2014	Reputation score 2016	Beds 2014	Beds 2016	Academic	Local Population 2014	Local Population 2015	GI faculty
Mayo Clinic	1	1	1,170,000	1,360,000	60.5	52.6	1132	1243	1	110,742	112,225	94
Cleveland Clinic	2	2	387,000	751,000	38.6	36	1268	1278	1	390,113	388,072	52
Massachusetts General Hospital	3	4	21,600	35,100	19.1	18.6	947	999	1	645,966	667,137	37
Johns Hopkins Hospital	4	3	301,000	392,000	24.3	23.5	951	998	1	622,104	621,849	46
UCLA^b^ Medical Center	5	5	19,300	25,000	11.1	8.6	466	466	1	3,884,000	3,971,883	44
Cedars-Sinai Medical Center	6	9	6655	9631	8.9	6.9	865	882	1	3,884,000	3,971,883	26
University of Pittsburgh Medical Center	7	6	6440	9246	13.3	9.2	1528	1517	1	305,841	304,391	66
New York Presbyterian Columbia and Cornell	8	14	22,300	28,200	7.7	5.7	2262	2328	1	8,406,000	8,550,405	84
Mount Sinai Hospital	9	7	46,300	63,400	16.5	11.8	1048	1183	1	8,406,000	8,550,405	63
Hospitals of the University of Pennsylvania	10	12	6746	9767	7.9	9.3	784	789	1	1,553,000	1,567,442	56
Northwestern Memorial Hospital	11	17	9533	13,100	6.8	5.7	885	885	1	2,719,000	2,720,546	53
Houston Methodist Hospital	12	11	1,1400	14,100	2.1	2	839	856	1	2,196,000	2,296,224	11
University Hospitals Cleveland Medical Center	13	27	10,600	13,700	1.6	2.3	771	790	1	390,113	388,072	27
Baylor University Medical Center	14	16	16,600	1680	4.6	3.4	876	844	1	1,258,000	1,300,092	40
St. Francis Hospital	15	19	136	304	0	0.1	306	306	0	2791	2791	32
Yale New Haven Hospital	16	23	13,800	17,400	2.6	2.6	1571	1576	1	130,660	130,322	48
Oschner Medical Center	17	27	3810	4905	1.9	1.6	771	789	1	23,319	23,319	10
Florida Hospital	18	—^c^	14,100	18,200	0.7	1.2	2338	2478	0	255,483	270,934	17
Beaumont Hospital	19	18	6113	7752	0.4	0.9	1070	1070	1	58,946	59,008	24
Lehigh Valley Hospital	20	38	6550	8847	0.6	0.1	793	784	0	118,577	120,207	22
St. Alexius Hospital	21	—	794	939	0.3	0	305	280	0	52,398	52,138	4
Brigham and Women's Hospital	22	45	21,100	29,000	4.1	5	779	757	1	645,966	667,137	44
University of Kansas Hospital	23	35	2331	3881	0.5	0.6	623	713	1	148,483	151,306	13
UCSF^d^ Medical Center	24	15	25,000	37,100	6.7	8.2	650	650	1	837,442	864,816	46
NYU^e^ Langone Hospitals	25	13	7697	12,100	1.9	2.4	791	718	1	8,406,000	8,550,405	54
University of Washington Medical Center	26	31	14,900	17,800	2.5	2.4	450	428	1	652,405	684,451	19
Hackensack University Medical Center	27	—	2627	3864	0	0.1	685	710	0	44,113	44,834	52
Bethesda North Hospital	28	—	1706	2523	0	0	375	342	0	297,517	298,550	19
John Muir Medical Center	29	—	0	207	0	0.1	367	383	0	66,900	68,910	29
Mayo Clinic Phoenix	—	8	1,170,000	1,360,000	—	—	—	268	1	1,513,000	1,563,025	27
Mayo Clinic Jacksonville	—	10	1,170,000	1,360,000	—	—	—	249	1	842,583	868,031	28
Thomas Jefferson University Hospital	—	19	—	9377	—	2.9	—	937	1	1,553,000	1,567,442	38
Stanford Health Care	—	21	—	24,000	—	3.5	—	481	1	66,642	66,853	47
University of Colorado Hospital	—	22	—	1518	—	2.3	—	648	1	345,803	359,407	19
University of Michigan Hospitals	—	23	—	21,900	—	6.5	—	962	1	117,025	117,070	51
Indiana University Health Medical Center	—	25	—	22,800	—	3	—	1243	1	852,866	853,173	76
Tampa General Hospital	—	26	—	8406	—	1.9	—	1011	0	352,957	369,075	18
Barnes-Jewish Hospital	—	28	—	7790	—	4.6	—	1323	1	318,416	315,685	23
University of Wisconsin	—	30	—	17,000	—	0.5	—	544	1	243,344	248,951	30

^a^GI: Gastrointestinal

^b^UCLA: University of California, Los Angeles

^c^Not applicable.

^d^UCSF: University of California, San Francisco

^e^NYU: New York University

**Table 2:** Multivariate analysis for 2014 Division Ranking per United States News and World Report.

Covariates	β coefficient	*P* value
Twitter followers	0.00004	<.001
Academic affiliation	5.3	.04
Total number of beds	0.0011	.59
Local population	–0.0000004	.58
Number of GI^a^ staff	0.066	.22

^a^GI: gastrointestinal

**Table 3:** Multivariate analysis for 2014 Reputation Score per United States News and World Report.

Covariates	β coefficient	*P* value
Twitter followers	–0.00002	.03
Academic affiliation	–7.3	.03
Total number of beds	–0.004	.12
Local population	–0.0000006	.51
Number of GI^a^ staff	0.042	.55

^a^GI: gastrointestinal

## Discussion

### Key Results

Based on the 2014 USNWR figures, both institutional Twitter following and academic affiliation are independent predictors of gastroenterology, and GI surgery subspecialty, rankings and reputation scores. Furthermore, gaining Twitter followers between 2014 and 2016 was significantly associated with improved or maintained divisional ranking, with 8000 more followers as the greatest predictor of improved or stable divisional ranking as noted on sensitivity analysis.

According to the Pew Research Center, social media usage has seen a nearly 10-fold increase over the past more than ten years since 2005, with 65% of all adults using a social networking site [13]. According to the Mayo Clinic Health Care Social Media List compiled in 2012, 1583/6562 hospitals in the United States have accounts on one or more social media platforms (eg, Twitter, YouTube, Facebook, LinkedIn, Foursquare), of which 1010 have Twitter accounts [14]. Thus far, gastroenterologist reception of the use of social media has been lukewarm, with only 57.5% of physicians perceiving social media as a method of obtaining “current, high-quality information” [15]. Only 47.7% of gastroenterologists reported having ever used any form of social media [16]. A better understanding of how social media might impact health-related and monetary outcomes might help galvanize use among medical professionals.

More importantly, many consumers use social media to access health-related consumer reviews, including hospital reviews [3,4]. Boosting a hospital’s social media presence could be a simple and economical way of potentially elevating institutional ranking, attracting more patients, and increasing hospital revenue. By using the principles of behavioral economics, hospitals would see that it seems that simplified ranking systems drive decision making for patients over complex measures of hospital quality [9,11]. The Twitter presences of United States universities, including an extended followers network, have been shown to reflect institutional rankings, like that of UNSWR [17]. Geotagged Twitter dialogue has also been shown to reflect population-level sentiments as well as disease states and changes by geography [18,19]. Pope et al report a shift from a lower hospital rank to a higher rank is associated with both an increase of nonemergency Medicare patients and an estimated $76 million in additional revenue [9]. Furthermore, rankings influenced an estimated 15,000 hospital choices made by Medicare patients over the course of a decade, resulting in a $750 million shift in revenue [9].

The academic community may question the validity and transparency of the USNWR rankings as an objective representation of hospital quality. One study found USNWR ranked institutions had reportedly demonstrated better clinical outcomes in cardiac care and mortality for acute myocardial infarction when compared to their unranked counterparts [20]. However, Hota et al noted discrepancies between certain patient safety indicators (PSIs), as established by the Centers for Medicare and Medicaid Services (CMS), and patient safety scores as reported in USNWR [21]. Since these concerns have been raised (after our analysis), USNWR have announced changes to their ranking methodology in June 2016 that include reducing the weight of the patient safety score and eliminating one of these discrepant PSIs [22]. Even with objective measures, an institutional ranking might not accurately represent the academic or technical capabilities of an individual practitioner. In nonobjective measures, our study also demonstrated significant associations between social media presence and reputation scores. Some researchers have also noted disproportionate influence of reputation score on subspecialty ranking over objective measures [23]. However, others have defended the objectivity of reputation score, citing strong correlations with research productivity, which could impact professional perception of a division [24]. Though our study demonstrated academic affiliation as an independent predictor of ranking, some hospitals may be ranked highly for good performance despite having no academic affiliation. The converse is also true, as there are large, reputable gastroenterology divisions with relatively robust social media presences that were unranked by USNWR at the time of this study.

### Limitations

The limitations of our study included using a single social media platform and using a single source for institutional rankings (USNWR), both of which may limit the generalizability of our results. Moreover, only institutional Twitter accounts were included in the study. At the time of data collection, there were too few gastroenterology division–specific accounts (eg, @Duke_GI) and subspecialty- or disease-specific Twitter accounts (eg, @UChicagoIBD, @UCLAIBD, among others). Furthermore, any causality cannot be directly established from our results. The differences in hospital rankings could also potentially affect social media following. Further research would therefore be required to elucidate the mechanism of how greater social media presence impacts hospital ranking, or if a higher divisional ranking may lead to a greater social media following. While reputation score determines 27.5% of the overall USNWR specialty score, there is also no existing data evaluating the number of Doximity voters who are active social media users or have significant exposure to dialogue occurring on Twitter [22]. Moreover, annual fluctuations in ranking might not reflect long-term social media impact, as previous studies have shown that hospital specialties vary by an average 5.49 spots per year [9]. There may have also been temporal differences that may have affected social media following at time of data collection, as data was collected at different times of year (June and November of either year). Finally, other factors could affect social media following but may not impact institutional rankings, such as significant news stories and exposure through other media outlets.

### Conclusion

Institutional social media following is independently associated with USNWR divisional ranking and reputation score. Moreover, an increase in social media following was also independently associated with improved or stable divisional ranking and reputation score with a threshold of 8000 additional followers as the best predictor of improved or stable ranking. Institutions hoping to boost their overall and divisional rankings may benefit from strengthening their social media presence by both engaging the public and increasing online visibility through platforms such as Twitter.

## Abbreviations

AUC: area under the curve
AUROC: area under the receiver operating characteristic curve
CMS: Centers for Medicare and Medicaid Services
GI: gastrointestinal
PSI: patient safety indicator
USNWR: United States News and World Report
