# Correction: Association Between Institutional Social Media Involvement and Gastroenterology Divisional Rankings: Cohort Study

**DOI:** 10.2196/29042

**Published:** 2021-03-29

**Authors:** Austin Lee Chiang, Loren Galler Rabinowitz, Akhil Kumar, Walter Wai-Yip Chan

**Affiliations:** 1 Brigham and Women's Hospital Boston, MA United States; 2 Harvard Medical School Boston, MA United States; 3 Columbia University Medical Center New York, NY United States; 4 Washington University in St Louis St Louis, MO United States

In “Association Between Institutional Social Media Involvement and Gastroenterology Divisional Rankings: Cohort Study” (J Med Internet Res 2019;21(9):e13345) the authors noted one error.

In the originally published paper, the name of author Loren Galler Rabinowitz was displayed incorrectly as surname “Galler Rabinowitz” and given name “Loren.” This has been changed to the correct format of surname “Rabinowitz” and given names “Loren Galler.” Accordingly, the citation for this author will be corrected from “Galler Rabinowitz L” to “Rabinowitz LG.”

The correction will appear in the online version of the paper on the JMIR Publications website on March 29, 2021, together with the publication of this correction notice. Because this was made after submission to PubMed, PubMed Central, and other full-text repositories, the corrected article has also been resubmitted to those repositories.

